# Editorial: Regulatory problems and disorders in early childhood: aetiology, contextual factors, developmental outcomes and pathways, and treatment options

**DOI:** 10.3389/frcha.2024.1534024

**Published:** 2024-12-20

**Authors:** Anna Katharina Georg, Julia Jaekel, Ayten Bilgin

**Affiliations:** ^1^ Institute for Psychosocial Prevention, University Hospital Heidelberg, Heidelberg, Germany; ^2^ Clinical Psychology and Psychotherapy of Childhood and Adolescence, University Tübingen, Tübingen, Germany; ^3^ Unit of Psychology, Faculty of Education and Psychology, University of Oulu, Oulu, Finland; ^4^ Department of Pediatrics, Essen University Hospital, Essen, Germany; ^5^ Department of Psychology, University of Copenhagen, Copenhagen, Denmark; ^6^ Department of Psychology, University of Warwick, Coventry, United Kingdom; ^7^ Department of Psychology, University of Essex, Colchester, United Kingdom

**Keywords:** early regulatory problems, early regulatory disorders, parent-infant interaction, parenting interventions, developmental trajectories, parenting stress

**Editorial on the Research Topic**
Regulatory problems and disorders in early childhood: aetiology, contextual factors, developmental outcomes and pathways, and treatment options

We are delighted to present a diverse selection of current research on early regulatory problems (RPs; i.e., excessive crying, sleeping, or feeding problems) and parent-child interactions. The articles in this special topic range from primary data studies of heterogeneous and diverse populations from Denmark, the USA, and Germany (both longitudinal, cross-sectional, and interventions), to meta-analytic approaches, and an expert opinion. After the last few decades empirically established a clear association of early RPs with risks for later childhood mental health (1, 2), we can now focus on the clinical and societal importance of this topic and underlying mechanisms.

## What risks do early regulatory problems pose to the child’s later mental health?

Several studies explore the question of whether and under which conditions early RPs place a child at risk for later mental health problems. The meta-analysis by Galling et al., provides a summary of studies conducted in clinical and community settings with follow-up ages ranging from 2 to 14 years. Pooled results show that children with RFs are 4 times more likely to develop overall behavioural problems than controls. Interestingly, children with multiple RPs are not at a higher risk than those with single RPs. Using different samples from Denmark, Ammitzboll et al. show that infants with RPs (as assessed by community mental health nurses) are at a higher risk of clinical diagnoses at 1.5 years. Similarly, Weber-Pant et al. show that the concerns of community mental health nurses about combined sleeping and feeding problems are associated with any neurodevelopmental disorder and autism spectrum disorder diagnoses at 1–8 years. Furthermore, Keller et al. investigate the impact of the COVID-19 pandemic on RPs and child mental health in a clinical sample. While parents do not report increased RPs during lockdown phases and the severity of pandemic restrictions have no impact, parental symptoms of depression are related to increased child behavioural problems in all age groups.

These studies support existing empirical evidence that RPs might reflect one of the earliest signs of an ongoing pattern of mental health problems that develop over time. In addition to the already mentioned robust evidence regarding the impact on later mental health problems, there are several arguments supporting this claim. First, the prevalence of RPs in infancy (approximately 20%) is similar to the overall prevalence of mental health problems in childhood. Second, consistency of the association between early RPs and mental health symptoms in childhood is demonstrated with evidence from a range of countries (e.g., Denmark, Germany, Finland, UK, Australia, Brazil) (3–9). Third, there is emerging evidence that RPs are associated with individual variations in the brain (i.e., default mode network) and physiological systems (i.e., the dysregulation of the HPA axis) that might account for the long-term negative impact on child mental health (10, 11).

## Parent-child interactions as a contextual driver of long-term consequences

Long-term effects of RPs are now well documented and underscore the need to better assist affected families. However, little is known about the potential underlying mechanisms explaining long-term consequences on mental health. In this regard, examining parent-child interactions in affected families may help understand the complex and dynamic interplay of biological and environmental mechanisms over time (12). This may require, as shown in this article selection, developing and adapting new parameters to address the specific characteristics of these interactions. For example, Licata-Dandel et al. demonstrate that mothers of children with RPs use more appropriate mind-related comments as well as non-attuned mind-related comments when interacting with their children than mothers in a control group. The results suggest that RPs are related to modified interactional processes of maternal-infant attachment. Accordingly, Jaekel et al. show that more persistent RPs in infancy are associated with lower quality of dyadic autonomic emotional co-regulation using the universal Welch Emotional Connection Screen (uWECS) coding system in a linguistically diverse sample. Together with the findings from Hane et al., these promising pilot study results facilitate a window into evolutionary-based parent-child co-regulatory processes that are not only related to RPs but also have an inherent function for our species and potentially contribute to long-term mental health outcomes. We encourage future research to build on these results and further explore the underlying biopsychosocial mechanisms, particularly in the context of stressful parent-child interactions and their explanatory power in terms of child developmental and mental health outcomes.

## How can we advance our understanding about regulatory problems?

Based on the identification of specific parameters of parent-child interactions and related mechanisms, we can finetune evidence-based intervention approaches (13). We already look back on effective treatment forms developed during the last decades (14, 15), but in some cases effect sizes are small and therefore unsatisfactory. In this topic selection, St James-Roberts and Llewellyn provide an expert opinion on the importance of providing support to parents of infants with RPs. Furthermore, two randomized control trials show that family-based interventions can have a range of benefits for developmental outcomes of preschool children with RPs (Welch et al.).

In addition to a clear need for large-scale assessments of evidence-based interventions and replication across different populations, another priority for future research includes the diagnostic specification of regulatory disorders, as there is currently no standardized approach. A unification of definitions and diagnostic methods would facilitate more comparable research as well as applied screening and prevention efforts. Finally, studies should systematically examine the implementation of interventions for early RPs to identify possible target-group specific barriers and facilitators to offer feasible and effective treatment options to affected families. We are enthusiastic that the selection of articles included in this special topic will contribute to progress in these areas and stimulate further research.
